# Depression in Alzheimer's Disease: A Delphi Consensus on Etiology, Risk Factors, and Clinical Management

**DOI:** 10.3389/fpsyt.2021.638651

**Published:** 2021-02-26

**Authors:** Luis Agüera-Ortiz, Rocío García-Ramos, Francisco J. Grandas Pérez, Jorge López-Álvarez, José Manuel Montes Rodríguez, F. Javier Olazarán Rodríguez, Javier Olivera Pueyo, Carmelo Pelegrin Valero, Jesús Porta-Etessam

**Affiliations:** ^1^Service of Psychiatry, Instituto de Investigación i+12, Hospital Universitario 12 de Octubre, Madrid, Spain; ^2^Centro de Investigación Biomédica en Red de Salud Mental (CIBERSAM), Madrid, Spain; ^3^Movement Disorders Unit, Hospital Clínico San Carlos, Complutense University, Madrid, Spain; ^4^Service of Neurology, Hospital General Universitario Gregorio Marañón, Complutense University, Madrid, Spain; ^5^Service of Psychiatry, University Hospital Ramón y Cajal, CIBERSAM, IRYCIS, University of Alcala, Madrid, Spain; ^6^Service of Neurology, HGU Gregorio Marañón, Madrid, Spain; ^7^Memory Disorders Unit, HM Hospitales, Madrid, Spain; ^8^Service of Psychiatry, Hospital Universitario San Jorge, Huesca, Spain; ^9^University of Zaragoza Associate Professor, Zaragoza, Spain; ^10^Service of Neurology, Instituto de Neurociencias, Hospital Clínico San Carlos, San Carlos, IdISSC, Madrid, Spain

**Keywords:** Alzheimer's disease, dementia, depression, prodromal symptoms, cholinesterase inhibitors, dual and multimodal antidepressants, precognitive action, consensus

## Abstract

**Background:** Alzheimer's disease (AD) and other forms of dementia are among the most common causes of disability in the elderly. Dementia is often accompanied by depression, but specific diagnostic criteria and treatment approaches are still lacking. This study aimed to gather expert opinions on dementia and depressed patient management to reduce heterogeneity in everyday practice.

**Methods:** Prospective, multicenter, 2-round Modified Delphi survey with 53 questions regarding risk factors (11), signs and symptoms (7), diagnosis (8), and treatment (27) of depression in dementia, with a particular focus on AD. The questionnaire was completed by a panel of 37 expert physicians in neurodegenerative diseases (19 neurologists, 17 psychiatrists, and 1 geriatrician).

**Results:** Consensus was achieved in 40 (75.5%) of the items: agreement in 33 (62.3%) and disagreement in 7 (13.2%) of them. Among the most relevant findings, depression in the elderly was considered an early sign (prodromal) and/or a dementia risk factor, so routine cognitive check-ups in depressed patients should be adopted, aided by clinical scales and information from relatives. Careful interpretation of neuropsychological assessment must be carried out in patients with depression as it can undermine cognitive outcomes. As agreed, depression in early AD is characterized by somatic symptoms and can be differentiated from apathy by the presence of sadness, depressive thoughts and early-morning awakening. In later-phases, symptoms of depression would include sleep-wake cycle reversal, aggressive behavior, and agitation. Regardless of the stage of dementia, depression would accelerate its course, whereas antidepressants would have the opposite effect. Those that improve cognitive function and/or have a dual or multimodal mode of action were preferred: Duloxetine, venlafaxine/desvenlafaxine, vortioxetine, tianeptine, and mirtazapine. Although antidepressants may be less effective than in cognitively healthy patients, neither dosage nor treatment duration should differ. Anti-dementia cholinesterase inhibitors may have a synergistic effect with antidepressants. Exercise and psychological interventions should not be applied alone before any pharmacological treatment, yet they do play a part in improving depressive symptoms in demented patients.

**Conclusions:** This study sheds light on several unresolved clinical challenges regarding depression in dementia patients. Further studies and specific recommendations for this comorbid patient population are still needed.

## Introduction

Aging is the strongest risk factor associated with dementia. Not surprisingly, with the aging of the world's population, the number of people living with dementia worldwide is expected to rise to 82 million in 2030 and almost double in 2050. Thus, considering that dementia is one of the most common causes of disability among the elderly, such estimates will have a physical, emotional and financial impact on dementia sufferers as well as their caregivers and relatives. The increased global economic and healthcare system burden cannot be dismissed either (1).

Chronic and progressive cognitive impairment is the clinical hallmark of dementias, namely Alzheimer's disease (AD) or other less common types such as vascular, Lewy body, and frontotemporal dementia (FTD) (1). Neuropsychiatric symptoms like depression often accompany and/or precedes dementia onset (1, 2). A recent meta-analysis determined that the prevalence of major depression was 15.9% and 14.8% in all-cause dementia and in AD, respectively (3). More strikingly, around one third of the adult population with depression is diagnosed with concomitant mild cognitive impairment (MCI) (4). In fact, it is thought that the presence of depression favors the conversion of MCI into AD later in life (5).

Given these known associations between depression and AD and the increasing rates of dementia, medical and community care services need to adjust to the specific needs and management of comorbid patients. However, depression in AD is still underdiagnosed and, therefore, undertreated most likely as a consequence of the lack of consistent diagnostic criteria to assess depression in this context (6). This, in turn, is challenged by the overlap of some symptoms. There are also discrepancies in reports between caregivers and patients, who tend to underestimate their symptoms of depression. On the other hand, depression recognition by caregivers varies depending on their level of stress and personal circumstances (7).

Once the diagnosis has been established, treatment regimens for depressed dementia patients are often extrapolated from clinical practice guidelines (CPG) or consensus on either AD or depression, which contributes to patient management heterogeneity (6). Besides, more controlled studies are needed to develop specific CPG recommendations for concurrent AD and depression. Only about a fifth of clinical trials of AD interventions considers neuropsychiatric symptoms like depression as a primary endpoint (8).

The aim of this Delphi study is to help homogenize the clinical care of patients with depression and dementia. Emphasis is placed on AD as it is the most frequent type of dementia. Since all consulted experts have broad experience in managing such patients, we expect to obtain specific diagnostic hints otherwise not included in current CPGs. We also suspect that prescription advice may not completely overlap with recommendations in the published guidelines for depression or dementia for this particular subgroup of patients for the reasons already mentioned.

## Materials and Methods

### Delphi Study Design and Methodology

This is a Modified Delphi study (9–11) based on a two-round closed-ended online survey. A total number of 53 items were grouped into 4 sections regarding risk factors (11), signs and symptoms (7), diagnosis (8), and treatment (12) of depression in AD and other dementias.

Participants responded to the 53 items of the questionnaire in the first round. Upon revision of the statistical results and comments made by panelists, they reconsidered the items for which consensus could not be reached in the second one. To do so, they anonymously assessed their level of agreement with every statement using a single ordinal 9-point Likert scale. A score value ranging from 1 to 3 was used to express disagreement (the lower the value, the stronger the disagreement); 4 to 6, half-way between agreement and disagreement (having a value of 4 demonstrated a tendency toward disagreement); and 7 to 9, agreement with the item (the higher the value, the stronger the agreement).

Consensus on each item was decided according to the RAND/UCLA criteria, i.e., when less than one third of the actual number of participants rates the item with a value outside the 3-point region which contains the median (9, 11, 13). For instance, given a median of 8, consensus agreement is obtained if <12 participants of this Delphi study (*N* = 37) rate the item outside the 7–9 region.

### Participants

The study was led by a Scientific Committee composed of 8 eminent physicians (4 psychiatrists and 4 neurologists) in the field of neurodegenerative diseases in the elderly. Duties assigned to the Committee were: Designing the study and protocol, writing the questionnaire, performing the statistical analysis, and analyzing and interpreting the results.

Although no formal sample size calculation is available for Delphi studies, a total number of 30–40 panelists was initially estimated as appropriate according to standard recommended practices (14). Finally, 37 physicians (27 men and 10 women) were invited and all medical specialties involved in the care of AD patients were covered (19 neurologists, 17 psychiatrists, and 1 geriatrist, which mirrors the distribution in the real-world clinical practice). All of them have a distinct curriculum in the field, were considered experts by their peers during the snowball selection process (15), and belong to tertiary hospitals with a spread geographical distribution throughout the country. Processing of personal information complies with all data protection and privacy laws and regulations.

### Statistical Analysis

Interpretation of the results was carried out using mean values and confidence intervals for the average at a 95% confidence level. Agreement or disagreement with each item depended on average values being closer to 9 or 1, respectively. Confidence intervals were informative of both unanimity of opinions and whether consensus agreement or disagreement could be reached for a given item. For instance, intervals containing the value 5, which corresponded to “neither agreement nor disagreement,” did not provide a consensus answer. In these cases, a descriptive reasoning was provided to participants.

## Results

**Figure 1:**
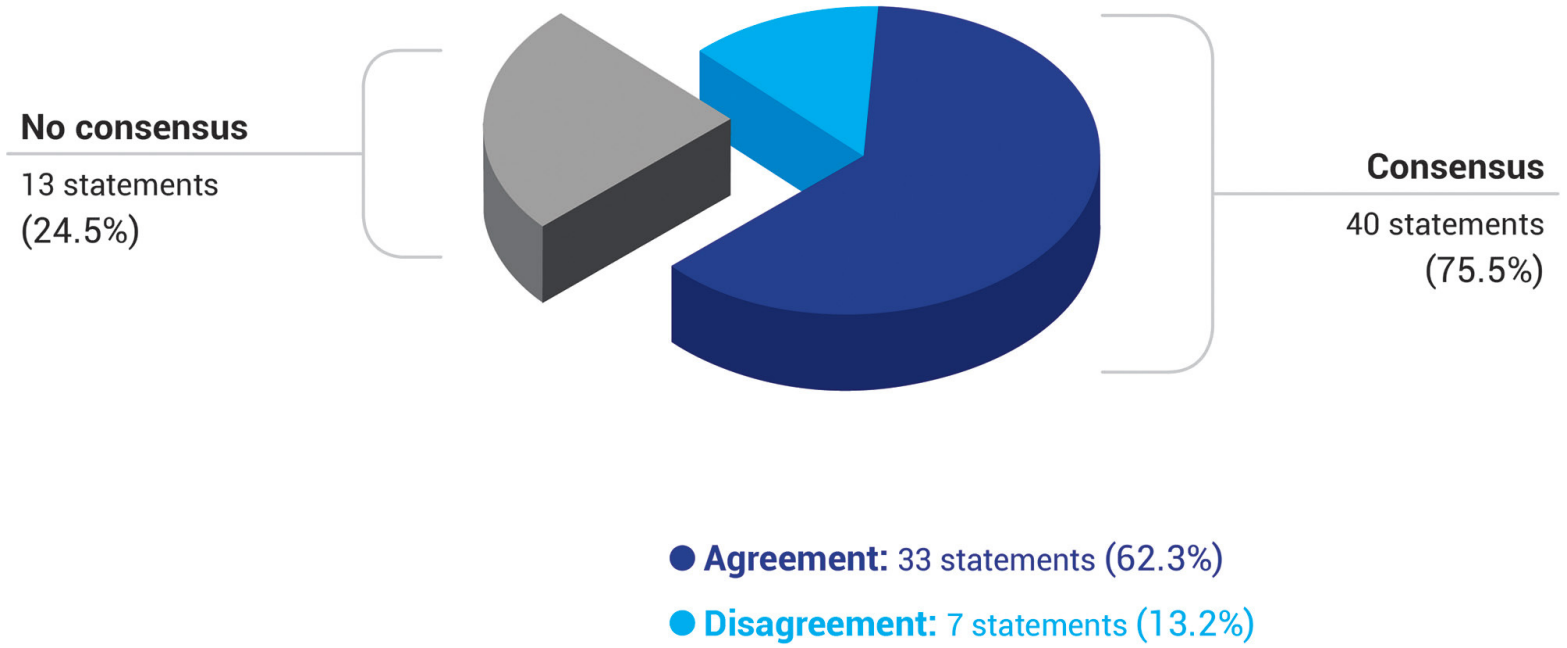
Degree of consensus, agreement, and disagreement among all participants of the Delphi study. *N* = 53 statements.

The Delphi survey was presented to the panel of experts in two successive rounds. In the first one, 33 (62.3%) items out of 53 reached consensus, rising to 40 (75.5%) in the second round. Overall, participants reached consensus to agree with 33 (62.3%) statements and to disagree with another 7 (13.2%). Approximately, one quarter of the questionnaire could not obtain consensus (13 items; 24.5%) (Table 1, Figure 1).

**Table 1:** Overall Delphi results.

**Statements**	**Consensus agreement**	**Consensus disagreement**	**Total consensus**	**No consensus**
First round, *n* (%)	33 (62.3%)	0 (0%)	33 (62.3%)	20 (37.7%)
Second round, *n* (%)	33 (62.3%)	7 (13.2%)	40 (75.5%)	13 (24.5%)
Section I—Etiology and risk factors for depression in dementia patients, *n* (%)	9 (16.9%)	1 (1.9%)	10 (18.9%)	1 (1.9%)
Section II—Clinical manifestations of depression in Alzheimer's disease and other dementias, *n* (%)	4 (7.6%)	0 (0%)	4 (7.6%)	3 (5.7%)
Section III—Diagnostic criteria for depression in Alzheimer's disease and other dementias, *n* (%)	5 (9.4%)	2 (3.8%)	7 (13.2%)	1 (1.9%)
Section IV—Antidepressant treatment for dementia and Alzheimer's disease patients, *n* (%)	15 (28.3%)	4 (7.6%)	19 (35.8%)	8 (15.1%)

*All statements. N = 53*.

Tables 2–5 show all Delphi statements and the resulting expert opinions toward each of them at the end of the study: No consensus or consensus, and if so, either agreement or disagreement. Items were classified into 4 categories attending to distinct clinical domains related to depression in AD. Regarding its etiology and potential risk factors, respondents agreed with most assertions [agreement in 9 items (16.9%), disagreement in 1 (1.9%), and no consensus in 1 (1.9%); Table 2]. The other domains were more controversial but agreement was still the predominant choice: Clinical manifestations [agreement in 4 items (7.6%) and no consensus in 3 (5.7%); Table 3]; diagnosis [agreement in 5 items (9.4%), disagreement in 2 (3.8%), and no consensus in 1 (1.9%); Table 4], and treatment [agreement in 15 items (28.3%), disagreement in 4 (7.6%), and no consensus in 8 (15.1%); Table 5] (Table 1).

**Table 2:** Section I—Etiology and risk factors for depression in dementia patients.

**Statement**		**Consensus agreement**	**Consensus disagreement**	**No consensus**
S1	Stress and/or depression in caregivers worsen symptoms of depression in patients with dementia	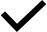		
S2	Social environment is determining for the onset of depression in dementia	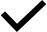		
S3	In the context of neurological diseases associated with dementia, depression is fundamentally reactive, and it depends on individual ways of becoming ill		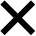	
S4	Late-onset depression increases the risk of dementia	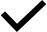		
S5	Depression that starts developing a few years prior to dementia onset constitutes a prodromal symptom and not an independent disorder	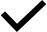		
S6	In elderly patients, depressive episodes accompanied by subjective memory loss complaints increase the risk of dementia	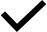		
S7	Greater vascular damage increases the risk of depression in dementia	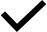		
S8	Treatment of depression in the context of dementia positively affects the course of the latter	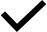		
S9	Disclosing the diagnosis of dementia to older patients increases the likelihood of clinically relevant depression			**—**
S10	Depression accelerates dementia progression	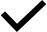		
S11	Given the link between depression and neurodegenerative diseases, patients over 50 with any symptoms of depression must be followed regularly, even if they show signs of improvement	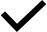		

**Table 3:** Section II—Clinical manifestations of depression in Alzheimer's disease and other dementias.

**Statement**		**Consensus agreement**	**Consensus disagreement**	**No consensus**
S12	The most specific symptoms of depression in AD are the ones that define the so-called somatic syndrome of depression (daily mood fluctuations, early-morning awakening, psychomotor retardation, or anorexia with weight loss)	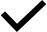		
S13	In late-stage dementia, symptoms of depression include sleep-wake cycle reversals, aggression, and agitation	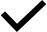		
S14	Apathy is a depression risk factor in dementia			**—**
S15	The existence of depression in AD patients undermines the neuropsychological assessment	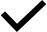		
S16	In advanced AD, the term “depression” should be replaced by “symptoms of depression”	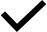		
S17	In depressive and dementia patients, autolytic behavior is only exceptional			**—**
S18	Depressive pseudodementia is a useful clinical concept and should be preserved.			**—**

*AD, Alzheimer's disease*.

**Table 4:** Section III—Diagnostic criteria for depression in Alzheimer's disease and other dementias.

**Statement**		**Consensus agreement**	**Consensus disagreement**	**No consensus**
S19	Information provided by the relatives is not very precise for the diagnosis of depression in dementia		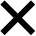	
S20	Sadness, depressive cognitions, and early-morning awakening distinguish depression from apathy in AD	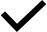		
S21	Structural neuroimaging is not useful for diagnosing depression in AD patients	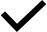		
S22	Functional neuroimaging (SPECT or, preferentially, brain PET) can be used to distinguish primary depression from depression secondary to AD			**—**
S23	Depression is a rare event in FTD		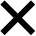	
S24	Differential depression diagnostic criteria are needed for the distinct diseases that can present with dementia (e.g., AD, PD, FTD, etc.)	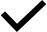		
S25	Differential depression diagnostic criteria are needed in the distinct stages of the dementia syndrome	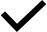		
S26	CSDD is a useful scale for detecting and assessing depression in older dementia patients in the daily clinical practice	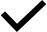		

*AD, Alzheimer's disease; CSDD, Cornell Scale for Depression in Dementia; FTD, frontotemporal dementia; PD, Parkinson's disease; PET, positron emission tomography; SPECT, single photon emission computed tomography*.

**Table 5:** Section IV—Antidepressant treatment for dementia and Alzheimer's disease patients.

**Statement**		**Consensus agreement**	**Consensus disagreement**	**No consensus**
S27	Physical activity improves symptoms of depression in dementia	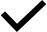		
S28	Antidepressants are efficacious drugs for treating depression in AD	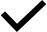		
S29	Combining antidepressants with ChEIs has a synergistic effect on the treatment of depression in AD patients	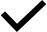		
S30	Tianeptine is an efficacious drug in AD patients with depression	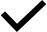		
S31	Psychological interventions like increasing social contact, reminiscence therapy, and cognitive rehabilitation are efficacious approaches to treating depression in dementia.	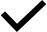		
S32	Non-pharmacological approaches to depression associated with dementia are the best initial option			**—**
S33	Patients suffering from both depression and AD should be treated with a ChEI before prescribing them any antidepressants		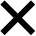	
S34	Patients suffering from both depression and AD should be treated with an antidepressant before prescribing them a ChEI			**—**
S35	Patients suffering from both depression and AD should be treated with an antidepressant and a ChEI at the same time			**—**
S36	Multiple-action antidepressants are the preferred choice for the treatment of depression in AD patients	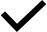		
S37	ChEIs have a positive effect on depression in AD	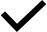		
S38	Memantine has a positive effect on depression in AD			**—**
S39	In patients with depression and cognitive decline, diagnosis of AD should wait until symptoms of depression and cognitive impairment have been followed upon antidepressant prescription	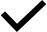		
S40	Antidepressants that improve cognitive function (tianeptine, vortioxetine, and duloxetine) are the preferred choice for the treatment of depression in AD patients	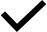		
S41	Treatment with antidepressants is less effective in older patients with AD than without it	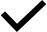		
S42	Antidepressants should be prescribed at lower doses in AD patients compared to non-cognitively impaired older patients		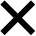	
S43	ECT is a recommended therapeutic option for the treatment of refractory depression in dementia patients			**—**
S44	TCAs are not to be used in patients with AD and depression	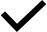		
S45	SSRIs are not to be used in dementia patients with depression and significant levels of apathy as they can worsen the latter			**—**
S46	Bearing in mind their specific efficacy and tolerability in AD, SSRIs are a good therapeutic option for depression in this context	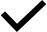		
S47	Bearing in mind their specific efficacy and tolerability in AD, dual and multimodal drugs are a good therapeutic option for depression in this context	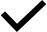		
S48	Bearing in mind their specific efficacy and tolerability in AD, mirtazapine is a good therapeutic option for depression in this context	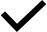		
S49	Bearing in mind their specific efficacy and tolerability in AD, bupropion is a good therapeutic option for depression in this context			**—**
S50	Bearing in mind their specific efficacy and tolerability in AD, nortriptyline is a good therapeutic option for depression in this context		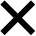	
S51	Bearing in mind their specific efficacy and tolerability in AD, agomelatine is a good therapeutic option for depression in this context			**—**
S52	Bearing in mind their specific efficacy and tolerability in AD, tianeptine is a good therapeutic option for depression in this context	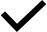		
S53	In general, the duration of treatment with antidepressants should be shorter in AD patients than in non-cognitively impaired older patients		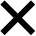	

*AD, Alzheimer's disease; ChEIs, cholinesterase inhibitors; ECT, electroconvulsive therapy; SSRIs, selective serotonin reuptake inhibitors; TCAs, tricyclic antidepressants*.

## Discussion

Research reports and medical guidelines specifically addressing depression in AD are scarce. Here, we present applicable expert opinions on the management of older individuals suffering or suspected to suffer from both clinical entities. The main limitation of the study is the local origin of panelists, which could reflect a country-specific approach. However, this also implies higher homogeneity among surveyed participants and therefore higher consistency of results, which reinforces the findings presented. Other limitations of the study are common with other Delphi studies, yet these are substantially restrained using the modified version, based on a two-round closed-ended survey. In short, in the Modified Delphi technique, items have already been pre-selected by a committee of experts based on their competent profile and revision of the available literature. Consequently, both the clinical relevance of questions addressed and consensus response rates are higher. Other assets are the possibility to offer controlled feedback to participants and assuring the anonymity of participants (11).

**Figure 2:**
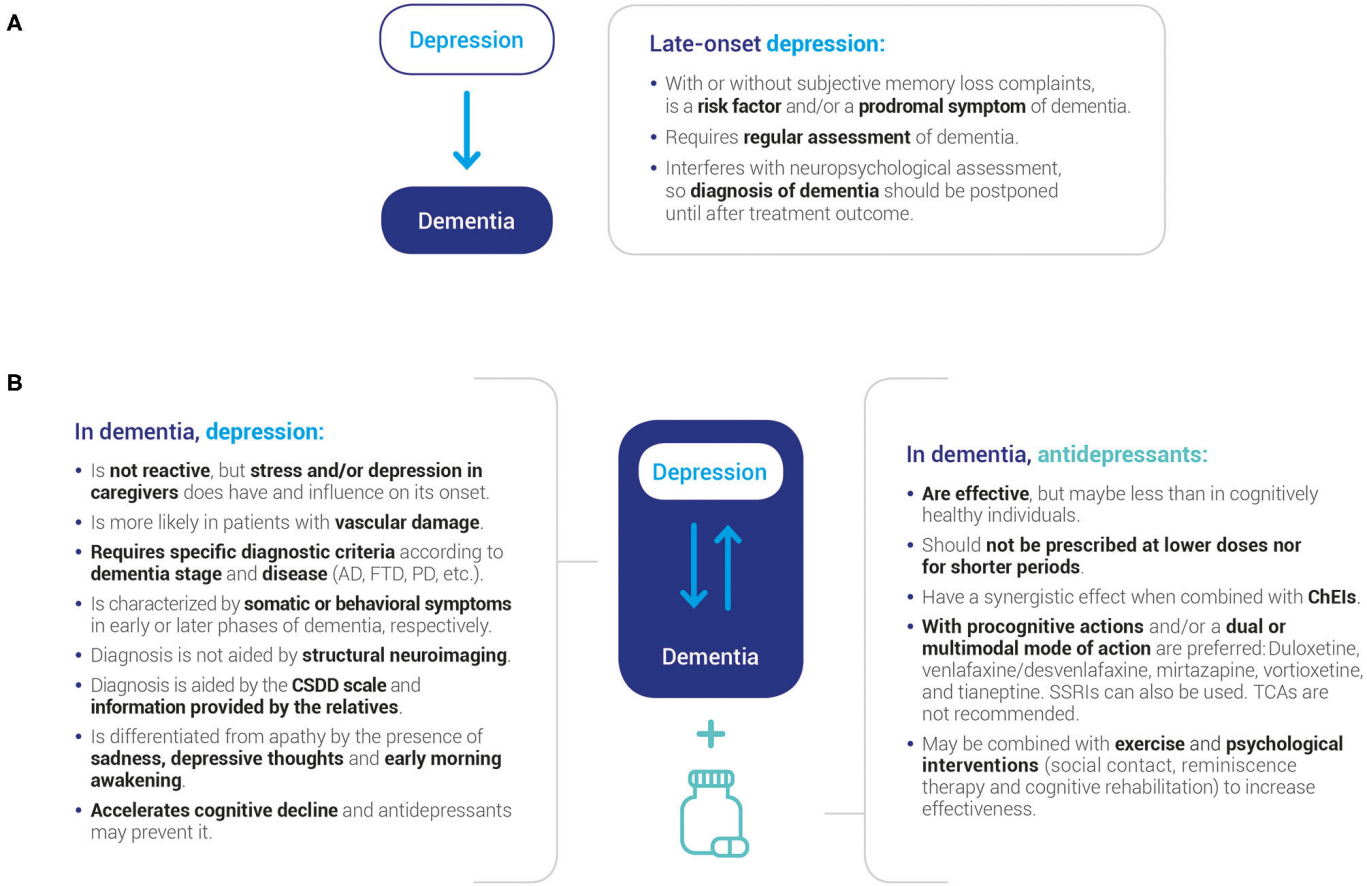
Key messages of the study. Consensus was reached on several fundamental statements regarding the relationship between late-onset depression and subsequent dementia **(A)** and depression in older individuals already diagnosed with dementia **(B)**. AD, Alzheimer's disease; ChEIs, cholinesterase inhibitors; CSDD, Cornell Scale for Depression in Dementia; FTD, frontotemporal dementia; PD, Parkinson's disease; SSRIs, selective serotonin reuptake inhibitor.

Due to the large number of items in the questionnaire, results are discussed in a question-answer format for ease of reading. Also, consensus and no consensus statements are contrasted with available depression, dementia, or mental health CPG recommendations as well as published evidence. Figure 2 shows the take-home messages of the present study, which may be used to inform clinical evaluation and aid decision making.

### I. Etiology and Risk Factors for Depression in Dementia Patients

#### Does Late-Onset Depression Favor Dementia Development?

There was consensus in all statements regarding late-onset depression and the likelihood of subsequent dementia (Table 2; S4, S5, and S11). Thus, the majority of respondents reckoned that depression that initiates in later phases of adulthood and into old age increases the risk of suffering from dementia (Table 2; S4). In the same line, experts came to the agreement that depression should be considered a prodromal symptom of dementia and not a stand-alone clinical entity (Table 2; S5). For this reason, a regular follow-up of depressed patients over 50 is paramount, even in the advent of symptom improvement (Table 2; S11).

The relationship between dementia and depression is supported by a growing body of evidence and the positioning of medical societies such as the European Federation of Neurological Societies (EFNS), the National Institute for Health and Care Excellence (NICE), the American Psychiatric Association (APA), and the World Federation of Societies of Biological Psychiatry (WFSBP) (16–21). Their respective CPGs recommend regular depressive symptom appraisal in elderly patients with dementia and assessment of other secondary causes (6), but these recommendations do not explicitly include following up on depressed patients at risk of dementia (i.e., before the diagnosis of dementia) to anticipate its onset.

Since the term “Behavioral and Psychological Symptoms in Dementia” (BPSD) was coined by the International Psychogeriatric Association (IPA) (2), many studies have demonstrated the existence of clinically-significant neuropsychiatric symptoms (including affective symptoms) prior to or over the course of dementia. Besides, the affective dimension has been included in the Mild Behavioral Impairment (MBI) diagnostic criteria and its associated MBI-Checklist (MBI-C) scale, which aims at improving the identification of patients at risk of dementia (22, 23).

Specifically, depression appears to be a dementia risk factor (cause) or an early sign (prodrome) of an underlying neurodegenerative disease typically associated with AD and other forms of dementia (12, 24–28). This interconnection seems to be dependent on age, depression severity, and success of antidepressant treatment (29, 30). In a large-cohort retrospective work by Barnes et al., the risk of dementia in patients with depressive symptoms greatly differs between midlife and late-life individuals (20% vs. 70%, respectively) (29). Results of another longitudinal study showed that the group of patients with a high-intensity and increasing depressive symptom trajectory were predisposed to develop dementia throughout the study period (more than a decade). This also underscores the importance of regular check-ups of patients with depressive symptoms over one-time assessments of depression in order to infer dementia risk (31).

#### Do Depressive Symptoms Worsen or Accelerate Dementia Progression?

In the context of neurodegenerative processes, experts agreed on the role of depression not only as a dementia initiation risk factor but as a progression enhancer (Table 2; S10). Conversely, treating the symptoms of depression that appear over the course of a neurodegenerative process would affect favorably its evolution (Table 2; S8).

Whether depressive symptom trajectory influences the progression of AD and other dementias has not been fully elucidated, and data from different authors have led to contradictory conclusions (32–35). However, there is mounting evidence to support common etiological mechanisms between depression and neurodegenerative pathologies (32, 36–38), therefore it seems plausible to suspect on an additive effect of depressive symptoms in AD. Genetic variations and neurobiological factors such as cerebrovascular disease, proinflammatory cytokines, cortisol and increased amyloid production, and accumulation may confer comorbidity risk (28, 37, 39). From a structural and functional point of view, a recent systematic review by Rashidi-Ranjbar et al. concluded that overlapping alterations in both frontal-executive and corticolimbic circuits, as well as alterations of global brain topology can be observed in many studies analyzing late-life depression and mild cognitive impairment patient samples (38).

There are also doubts as to whether antidepressants can reverse the deleterious effects of depression on dementia due to the scarcity of specifically driven studies. Long-term use of selective serotonin reuptake inhibitors (SSRIs) may delay dementia development in patients with MCI and depression (40). However, a previous meta-analysis had calculated a 2-fold increased risk of suffering from cognitive impairment or Alzheimer's dementia upon antidepressant drug usage, especially if this is started before age 65 (41), however this effect may be linked to depression itself rather than its treatment.

#### What Is the Clinical Relevance of Subjective Memory Loss Complaints in Depressed Older Patients?

The co-occurrence of depressive episodes and subjective memory loss complaints was also considered a dementia predisposition factor by the panel of experts (Table 2; S6).

In connection to this consensus statement, it is known that depression favors the transition from normal cognition to MCI, and from MCI to dementia (5, 42–44). In fact, around 30% of older adults with depression are also diagnosed with MCI (4). For this reason, repeated cognitive assessment in already diagnosed depressed patients, and especially in patients with depressive pseudodementia, is strongly supported by experts and some available published evidence as subsequent deterioration during the course of depression constitutes a prognosis factor and a clinical marker for dementia onset (24, 45). Nevertheless, some caution must be taken on such cognitive evaluation since depression itself may interfere with it (Section II. Clinical manifestations of depression in Alzheimer's disease and other dementias).

#### What Factors Are Involved in the Etiology of Depression in the Context of Dementia?

Depression that arises in the context of dementia was not considered a purely emotional reactive mental disorder (Table 2; S3), however there was consensus on the importance of social environment to its onset (Table 2; S2), including stress and/or depression in caregivers, which was believed to enhance their depressive symptomatology in a vicious circle (Table 2; S1). Consensus was also achieved on the pernicious effect that vascular damage has on the affective domain of these patients (Table 2; S7). On the contrary, there was no consensus on whether apathy also contributes to depression too (Table 3; S14).

As mentioned earlier, both disturbances appear to be linked through common neurobiological mechanisms (32, 36–39), but it is still unclear whether depression is an etiological factor, a prodromal symptom, a coincidental finding or a secondary effect of dementia (12, 27, 28). Thus, manifestations of depression may not precede cognitive decline but arise in the presence of clinically overt dementia (46, 47).

In this scenario, and according to the results presented here, neither environmental stressors nor the personal way in which an individual becomes sick play a central role in depression development in dementia compared to cognitively healthy individuals (Table 2; S3). Instead, neurobiological considerations related to the dementing disorder may be more preponderant here. As such, pathophysiological events such as increased vascular load and/or neurodegenerative processes may lead to structural and functional brain damage, thereby jeopardizing cognitive function and the affective domain (37, 46, 48, 49). There are, nonetheless, several studies that call into question the “vascular depression hypothesis,” i.e., depression secondary to cerebrovascular disease (50, 51).

As discussed later on Section III (Diagnostic criteria for depression in Alzheimer's disease and other dementias), comorbid apathy in dementia patients requires differential diagnosis as an independent clinical entity. Although being highly frequent in AD patients with depression, it can be present in non-depressed patients too. Interestingly, previous reports have shown that the frequency of apathy (but not depression) increase in time as dementia progresses (52, 53). Altogether, these data suggest that apathy in dementia may not predispose or be linked to depression.

#### How Important Is Disclosing the Diagnosis of Dementia to Older Patients in Regard to Depression Risk?

This is a controversial subject and such a question could not reach consensus among experts (Table 2; S9).

Whether the diagnosis of dementia should be disclosed to patients or not has triggered much ethical debate as they are exposed to the unpleasant reality of suffering from a degenerative disease with no breakthrough therapeutic options. As discussed in the next section (Section II. Clinical manifestations of depression in Alzheimer's disease and other dementias), some authors have suggested that sharing the diagnosis with them would increase suicidal risk (54, 55). Other studies suggest though that self-awareness of cognitive and functional impairment is only associated with dysthymia but not major depression (7). From this unresolved controversy and the lack of consensus in this Delphi questionnaire it follows that physicians should be encouraged to individually assess the best way and moment to deliver such information to the patient.

### II. Clinical Manifestations of Depression in Alzheimer's Disease and Other Dementias

#### Is the Concept of Depressive Pseudodementia Clinically Useful?

No consensus could be reached regarding the clinical utility of the concept of “depressive pseudodementia,” i.e., the presence of important cognitive symptoms inherent to depression, especially in the elderly (Table 3; S18) (56).

These inconclusive results may mirror the general skepticism surrounding the appropriateness of the term and its clinical value (45). In support of it, and as already stated before, depression is often involved in the progression of MCI into full-blown dementia (5, 42–44), so depressive pseudodementia can have prognostic implications in depressed patients who complain about subjective memory loss. Also, the use of this term can aid differential diagnosis as depression-related cognitive symptoms (pseudodementia) may be confused with dementia and produce false positives (45).

#### Does the Presence of Depression Interfere With Neuropsychological Assessment in AD Patients?

Experts agreed that symptoms of depression undermine neuropsychological test results in patients with AD (Table 3; S15) and so neuropsychological assessment should wait until antidepressant treatment outcomes can be observed in regard to affective and cognitive symptoms (Table 5; S39).

In accordance with the previous question, frontal and prefrontal executive deficit is the main cognitive symptom specifically attributed to depression in the elderly over memory, language, and visuospatial impairment (45). Thus, low cognitive performance in depressed adults can be an artifact for AD diagnosis. For this reason, it seems convenient to postpone it until adequate treatment of depression has been applied and patient's symptomatology has been followed over time: Absence of cognitive improvement upon depressive symptom remission would pinpoint an ongoing neurodegenerative process; a partial cognitive recovery would be indicative of either a partial depression remission or a certain degree of neurodegeneration, whereas a complete cognitive remission would correspond to a previous pseudodementia diagnosis with no added dementia.

#### Which Are the Most Specific Signs and Symptoms of Depression in AD?

Respondents agreed that the specific clinical manifestations of depression in early AD are the ones under the umbrella of the so-called somatic syndrome, which is typically found in the general adult population with depression: Daily mood fluctuations, early-morning awakening, psychomotor retardation, and anorexia with weight loss (Table 3; S12). In contrast, as the course of AD advances, the somatic syndrome is replaced by behavioral symptoms such as aggression, agitation, and reversal of the sleep-wake cycle (Table 3; S13). Current CPGs fail to provide clear and specific diagnostic guidance for this subset of patients (16–20, 57, 58).

#### Is It Appropriate to Use the Term “Depression” in Older Patients With Advanced AD?

In clear connection with the question above, experts unanimously recommended talking about “symptoms of depression” instead of “depressive disorder” (Table 3; S16) due to the lack of depression-specific diagnostic criteria in the later stages of AD (Section III. Diagnostic criteria for depression in Alzheimer's disease and other dementias).

#### Is Depression Commonly Reported in Other Less Frequent Types of Dementia?

When asked about FTD patients, respondents refused the idea that depression is a rare symptom in these cases (Table 4; S23).

In line with these results, a very recent systematic review on neuropsychiatric symptoms in different types of dementia found that, together with AD, FTD shows the highest prevalence of depression (59). Apart form that, given the almost absent cognitive symptom signature, initial clinical presentation of FTD may be confused with late-onset and primary psychiatric illness. In fact, MBI was originally described by Taragano et al. as early manifestations of FTD and, although they can be prodromal symptoms of other forms of dementia, patients who present with them (especially the ones without cognitive symptoms) are more prone to develop FTD than AD (22, 60, 61).

#### Is Autolytic Behavior Less Frequent in Dementia Patients With Comorbid Depression?

Consensus of opinion on the exceptionality of autolytical behavior in these patients could not be generated (Table 3; S17).

This is a much-disputed issue probably because of the existence of contradictory data. Some studies did not support a significative relationship between dementia and autolytic attempts (62). Others did identify an association between dementia and suicidal ideation, attempt and/or completion, especially soon after dementia diagnosis acknowledgment (54, 55, 63–65), which supports the idea that self-awareness of functional decline increases suicidal risk and/or patients with deteriorated brain function are more prone to catastrophic outcomes. In this line, APA guidelines for depression consider that patients with depression in dementia should be assessed for suicidality (20). Other factors have been associated with suicidal risk in dementia such as hopelessness, preserved insight, younger age, white race, previous inpatient psychiatric hospitalization, antidepressant and anxiolytic prescription, and resistance to anticholinesterases (54, 55).

### III. Diagnostic Criteria for Depression in Alzheimer's Disease and Other Dementias

#### Are the Same Depression Diagnostic Criteria Applicable to all Dementias or to Distinct Dementia Stages?

Experts agreed on the need to establish specific depression diagnostic criteria for the different entities that can present with dementia (AD, Parkinson's disease, FTD, etc.) as well as the stages of the dementia syndrome (Table 4; S24 and S25) because of the diverse repertoire of symptoms that varies according to the degree of neurodegeneration (Table 3; S12 and S13).

Other authors have also claimed the urge to define consensus criteria to diagnose depression in Alzheimer's and other dementias as it is currently underdiagnosed (7, 66). In the absence of specific biomarkers for the daily practice, such diagnosis is exclusively based on psychiatric interviews assessing a variety of emotional and behavioral symptoms which then meet certain pre-established criteria, such as those proposed by the National Institutes of Mental Health (NIMH) and published in 2002 (67). However, they remain somewhat unknown and not regularly used by healthcare professionals outside Psychogeriatrics. In contrast to the Diagnostic and Statistical Manual of Mental Disorders-IV (DSM-IV) criteria for major depression in adults, NIMH criteria for depression in AD (NIMH-dAD) require the presence of at least 3 symptoms (and not 5) out of a possible list of 10. This symptom list also contains some modifications in comparison with the DSM-IV criteria such as irritability and social isolation replacing loss of libido and loss of pleasure upon social contact instead of loss of interest (7).

#### Is It Possible to Differentiate Depression From Apathy in AD Patients?

As agreed during this study, sadness, depressive cognitions, and early-morning awakening are typically attributed to depression in dementia and not apathy (Table 4; S20).

Apathy is the most prevalent comorbidity associated with depression in AD. In the Cache County study, 27% of AD patients suffered from apathy and, of them, 40% also had depression (7). Despite the high co-occurrence, apathy constitutes an independent clinical entity from depression and has specific clinical criteria (68, 69). Apart from the consensus symptoms found in the present study, feeling of guilt, low self-esteem and hopelessness are more common in depression than in apathy (7).

#### Can Neuroimaging Ease the Diagnosis of Depression in AD?

Experts discarded structural neuroimaging techniques as useful diagnostic resources (Table 4; S21) but collective opinion on functional neuroimaging was not straightforward (Table 4; S22).

To date, there are no helpful biomarkers available to diagnose depression in the presence or absence of dementia, yet structural neuroimaging may be a valuable tool in ruling out the organic origin of depression. One of the main criticisms of single photon emission computed (SPECT) or brain positron emission (PET) tomography is that they lack enough specificity (70).

Despite this, recent evidence does pave the way for the use of such techniques and distinct surrogate biomarkers for depression in AD patients. In their voxel-based morphometry analysis, Karavasilis et al. suggested that Alzheimer and depression comorbidities may be characterized by a specific pattern of gray matter loss that coincides with anatomical regions of the sensorimotor system and the right thalamus (71). In the same line, and as discussed earlier on, Rashidi-Ranjbar et al.'s work focused on two neuroimaging techniques, namely diffusion-weighted imaging measuring white matter tract disruptions and resting-state functional MRI, to provide important clues on both functional and structural abnormalities shared by patients with late-life depression and/or MCI (38). Other studies show that [18F]-fluorodeoxyglucose (FDG) and amyloid-PET techniques allow *in vivo* assessment of regional glucose metabolism and beta-amyloid plaque deposition, respectively, and their association with cognitive, behavioral, and physical performance in MCI and AD (72, 73).

#### Are There Other Useful Approaches to Diagnosing Depression in Dementia?

Experts concurred that relatives can make a difference in refining the diagnosis of depression in dementia (Table 4; S19) and that the Cornell Scale for Depression in Dementia (CSDD) is useful for screening patients in everyday clinical practice (Table 4; S26).

Older adults tend to minimize and rationalize their behavioral and emotional symptoms by linking them to specific situations or the mere aging process, yet depression is not a *sine qua non* clinical feature of the latter. This biased normalization may hinder the diagnosis of depression during patient interviews. Therefore, data gathered from relatives and caregivers can aid the task and complete the clinical information.

Among the plethora of available depression scales, CSDD appeared to be the preferred one to detect and assess the severity of depressive symptoms in older dementia patients (Table 4; S26), which matches common recommendations from APA, EFNS and the Institute for Clinical Systems Improvement (ICSI) guidelines (16, 19–21). Other scales like the Geriatric Depression Scale (GDS) or the Dementia Mood Assessment Scale (DMAS) may be used in these cases, but CSDD shows the highest performance. Moreover, it is a clinician-rated scale that involves caregivers, which is an asset in this clinical context as it allows bypassing of the aforementioned patient bias (6).

### IV. Antidepressant Treatment for Dementia and Alzheimer's Disease Patients

#### Is Antidepressant Medication Effective in Treating Depression in Elderly Patients With AD?

Consensus could be reached comfortably in regard to the efficacy of antidepressants for this indication (Table 5; S28), although it was also agreed that it is less effective in dementia patients than in the absence of cognitive problems (Table 5; S41). Regarding the treatment regimen, experts rejected prescribing both lower doses of antidepressants and shorter treatment periods in AD patients than in cognitively healthy patients (Table 5; S42 and S53).

Pharmacological therapy for depression in dementia is recommended by most CPGs analyzed with some variations (17–19, 57, 74). Whereas the World Health Organization (WHO) guidelines consider that psychiatric drugs are warranted in cases of moderate-severe depression (74), NICE emphasizes the need to assess the risk-benefit ratio on a case-by-case basis (17). Others such as the Fourth Canadian Consensus Conference on the Diagnosis and Treatment of Dementia (CCCDTD4) recommends drug therapy if non-pharmacological treatment fails (57).

Among several reasons behind the disparity of results from drug trials, it is interesting to mention the existence of auto-limited cases of depression that ultimately resolve without the need for treatment, which may justify the high placebo effect observed in AD trials (75, 76). Also, some studies show that antidepressant drug response varies upon cognitive performance scores, whereas others do not support such a claim (8, 77). Despite this, it follows from this consensus that treatment nihilism should not be an option in dementia patients, even if initial antidepressant resistance occurs.

The fact that AD patients usually have several medical comorbidities and therefore are exposed to polypharmacy may lead to dosage adjustments compared to dementia-free adults. Here, experts declared themselves against such principles (Table 5; S42 and S53) to avoid the use of infratherapeutic doses and the occurrence of treatment pseudoresistance.

#### Should Anti-dementia Cholinesterase Inhibitors Be Used to Treat Depression in Alzheimer's Patients?

Experts acknowledged the positive effect of cholinesterase inhibitors (ChEIs) on depressive symptoms and their putative synergism with antidepressants to treat depression in the context of AD (Table 5; S29 and S37). However, the order in which they should be administered could not be clarified (Table 5; S33 and S35).

In general, scarcity of trials dedicated to pharmacological alternatives for depression in dementia seems the most plausible explanation for a lack of consensus on these items of the questionnaire. Add-on ChEIs in depressed older patients do not seem to be more effective than antidepressants alone (78–80), although one study suggests the opposite (81). Here, combination therapy may have been disregarded due to safety concerns (drug-drug interactions). For instance, anticholinergic antidepressants may antagonize the effect of ChEIs (82), so concurrent use of both drugs seems like a futile and unsafe therapeutic strategy, especially in dementias that are characterized by cholinergic deficiency such as AD or Lewy Body Disease. In relation to this, CPGs highlight the significance of anticholinergic side effects when choosing an antidepressant for dementia patients (16, 17, 19, 20, 57).

Regarding the benefits of ChEIs in depression (Table 5; S37), the British Association for Psychopharmacology and several reports suggest that ChEIs may ameliorate neuropsychiatric symptoms in Alzheimer's or Lewy body dementia (58, 83–85). Since the etiology of depression seems to be linked to the neurodegenerative process, any improvements of the latter may also be advantageous for depressive symptomatology.

#### Should Memantine Be Prescribed in Depressed Alzheimer's Disease Patients?

Apart from its indication for moderate to severe AD, no consensus could be reached on the advantages (if any) of memantine in depression treatment in these patients (Table 5; S38).

Coadjuvant treatment with antidepressants and memantine showed good depressive and cognitive outcomes in patients with cognitive decline (86, 87). However, more studies are needed to analyze whether combining ChEIs or memantine with antidepressants has any added cognitive, affective, or functional benefits.

#### Which Antidepressant Drug Groups Are More Appropriate for Depressive Symptoms in Dementia?

In dementia, antidepressants with multiple mechanisms of action (dual agents like venlafaxine/desvenlafaxine or duloxetine, mirtazapine as well as tianeptine or the multimodal vortioxetine) were preferred over others by this panel of experts (Table 5; S30, S36, S47, S48, and S52). Unimodal SSRIs were considered efficacious and well-tolerated drugs for this indication (Table 5; S46), whereas tricyclic antidepressants (TCAs), including nortriptyline, were not recommended (Table 5; S44 and S50). Agomelatine and bupropion could not reach such a consensus (Table 5; S49 and S51).

SSRIs are still the first-choice antidepressants in adult patients with any comorbidities and in geriatric depression (47, 88). Some studies point toward a higher effectiveness of the SSRI sertraline compared with other antidepressants regulating more than one neurotransmitter pathway (89), others found no advantages for sertraline, fluoxetine, or mirtazapine vs. a placebo in cognitively impaired patients with depression (75, 90). As suggested by Lozupone and other experts, methodological discrepancies in randomized controlled trials may be behind the disparity of results among the few studies on antidepressant treatment in AD (91), which adds to the challenge of establishing clear recommendations. Prescription of SSRIs is also questionable in other clinical conditions such as apathy. SSRIs and other medications can have an apathogenic effect (92), so although consensus could not be obtained regarding their use in patients with significative apathy (Table 5; S45), it may be advisable to monitor the symptoms and reduce the dose and/or replace them with a non-SSRI alternative accordingly.

Most CPGs state that SSRIs (citalopram or sertraline) and TCAs have similar efficacy, but SSRIs are preferred to avoid anticholinergic side effects (6). Here, TCAs were not recommended by the panel either, due to safety concerns. Nortriptyline was not an exception, although it appears to be the safest of its class (93). This consensus agreement matches not only CPG recommendations but several other published criteria for drug prescription in the elderly like the Beers and STOPP-START criteria (6, 94).

Along with SSRIs, consulted CPGs also recommend other drugs like bupropion, venlafaxine, and mirtazapine, which shows that there is no preference of one specific drug group over others (6). This contrasts with the opinion of the experts in the present study in favor of dual or multimodal antidepressants, especially venlafaxine/desvenlafaxine, duloxetine, mirtazapine, and vortioxetine. In a previous consensus on depression in the elderly, experts had also agreed on the superior efficacy of dual-action antidepressants compared to SSRIs in geriatric depression (47). In fact, it has been proposed that a subgroup of dementia patients with depression dominated by both affective and psychological symptoms (pessimism and low self-esteem) could benefit the most with mirtazapine. This subgroup of patients did not present with sleep problems, which seems counterintuitive since mirtazapine, with sedative properties, is more effective in patients with such condition (95).

Specific opinions on bupropion and agomelatine among participants were not as optimistic. We hypothesize that this may be due to increased risk of convulsions associated with bupropion uptake. However, its non-serotonergic and rather activating effects (96) would make it suitable for AD, where more noradrenergic symptoms prevail, or in the apathy syndrome in AD. No evidence could be found to explain the lack of consensus toward agomelatine, so we can only speculate on cost-associated causes or clinical habits.

#### Are Antidepressant Drugs With Precognitive Actions the Treatment of Choice in Alzheimer's Disease?

Experts unanimously considered that the gold standard antidepressant drugs in AD are the ones that improve cognitive function in geriatric depression (Table 5; S40). Of them, dual (duloxetine) and tianeptine as well as multimodal agents (vortioxetine) show a good efficacy and tolerability profile (Table 5; S30, S47, and S52).

Over recent years, awareness of the anticholinergic side effects of paroxetine and TCAs has increased as they compromise cognition (93). This becomes an issue of greater importance in cognitively impaired and dementia patients. Because of that, much attention has been drawn to the use of the antidepressant drugs with precognitive actions.

The number of studies addressing the efficacy and safety of these drugs in depression and dementia is still limited. A meta-analysis of 3 controlled trials concluded that vortioxetine, aside from its efficacy in depression, significantly improves cognitive function compared with duloxetine (97). An observational study in AD found significative cognitive advantages with vortioxetine in contrast to other antidepressants. However, the control group of the comparative arm was very heterogeneous and vortioxetine's efficacy was not directly tested against other drugs with precognitive actions like tianeptine (98).

Unlike other antidepressants, tianeptine has anxiolytic properties and can also improve somatic symptoms (99). In depression and AD, it shows higher tolerability and, consequently, higher patient compliance than other agents such as fluoxetine (100).

#### Should Non-pharmacological Approaches Be Considered in Depressed and Dementia Patients?

Experts reckoned that both physical activity and psychological interventions (social contact, reminiscence therapy, and cognitive rehabilitation) have antidepressant efficacy (Table 5; S27 and S31), yet this may not be enough as to warrant their application as an initial approach in dementia (Table 5; S32).

Some results from previous meta-analysis support expert opinion collected through this Delphi questionnaire but others point in different directions. The one published in 2015 by Forbes et al. found that evidence of the effects of physical activity on depression is inconclusive (101). Another one from 2019 suggested it may be beneficial for cognition and activities of daily living (ADL) of older individuals with dementia, but depression levels did not change (102). Conversely, another systematic review and meta-analysis released the same year did find a positive effect of home-based exercise on neuropsychiatric symptoms of dementia, ADL and physical fitness of older adults with dementia living at home as well as a reduction in caregiver's burden (103).

Similar to antidepressant drugs, it is worth mentioning that physical exercise cannot be regarded as a uniform whole, so that efficacy and safety assessment is a challenging issue that may explain discrepancies among studies. The same applies to other types of non-pharmacological options such as reminiscence therapy, which has significant scientific support in AD-related depression (104). Some CPGs like APA's and NICE's also recommend the inclusion of a variety of non-pharmacological options like the ones already mentioned or cognitive behavioral, animal-assisted, stimulation-oriented, or multi-sensory stimulation therapy (6).

Although experts recognized their efficacy, specific knowledge gaps and/or limited availability of non-pharmacological approaches may deter them from using them in the first place. On one hand, other experts proposed that these patients should be managed by non-pharmacological means initially unless presenting with severe depression or an inability to receive such therapies (105). On the other hand, CHROME criteria state that there are several reasons why psychiatric drugs should be applied from the beginning such as the previously observed good response to them or having a personal history of severe and recurrent depressive episodes (106).

#### Is Electroconvulsive Therapy an Acceptable Therapeutic Choice in Dementia?

Electroconvulsive therapy (ECT) could not be recommended nor rejected as a valid alternative for antidepressant refractory cases (Table 5; S43).

ECT is widely used in severe and drug-resistant cases of depression (47) and APA guidelines claim its use in certain cases of depression in those with dementia (20). Non-psychiatrist physicians may be unfamiliar with it, which could explain the larger proportion of neurologists in the panel in disagreement with this technique (data not shown). In spite of the existence of some cognitive adverse effects in the first 3 days post-ECT, meta-analytical data from depressed patients demonstrated that working and anterograde memory as well as processing speed (among other executive functions) improved after 15 days compared with baseline levels (107).

## Conclusions

An *ad-hoc* questionnaire was designed to gather opinion from expert physicians working in the field of neurodegenerative disease. Consensus agreement could be reached for most of the items addressing risk factors, clinical parameters, diagnostic criteria, and treatment of depression in AD and other dementias (Figure 2).

Depression that initiates in older adults, with or without subjective memory loss complaints, was considered an early sign (prodromal) and/or a dementia predisposition (risk) factor. Therefore, routine check-ups of depressed patients over 50 should be performed. Conversely, in already diagnosed dementia patients, experts agreed that depression would not only be an emotionally reactive mental disturbance related to the experimented impairment, although the influence of social environment on its onset cannot be dismissed. Instead, other neurobiological factors such as vascular damage would play a more prominent role. Once dementia is established, it was suggested that depressive episodes would accelerate dementia progression whereas antidepressants would have the opposite effect.

According to expert opinion, the existence of cognitive symptoms specifically related to depression undermines neuropsychological assessment in already demented patients. Also, caution should be taken when confirming the diagnosis of dementia, which should be postponed until treatment with antidepressants has been initiated and both depressive and cognitive symptoms have been followed for a reasonable period of time.

In advanced dementia patients, referring to “symptoms of depression” seemed more appropriate than the term “depression” or “depressive disorder” because there are no formal diagnostic criteria for depression in late-stage dementia. As observed in clinical practice, experts remarked that symptoms in such stages of dementia include reversal of sleep-wake cycle, aggressive behavior, and agitation. In contrast, depression in early dementia is characterized by somatic symptoms as in the general adult population: Daily mood fluctuations, early-morning awakening, psychomotor retardation, or anorexia with weight loss. Also, feeling sad, having depressive thoughts, and waking up early in the morning were found to be suggestive of depression and not apathy. To aid diagnosis of depression in dementia sufferers, CSDD scale and information from relatives were found useful, whereas structural neuroimaging techniques were discarded.

Unanimous consensus could be reached on the efficacy of antidepressants, although it may be lower than in cognitively healthy patients. Also, neither the dose nor the treatment period should be necessarily different in dementia patients. Anti-dementia ChEIs may have a positive effect on depressive symptoms and a synergistic effect together with antidepressants. However, their recommended order of administration remained unclear.

In AD, antidepressants of choice were those that improve cognitive function and/or have a dual or multimodal mode of action, namely duloxetine, venlafaxine/desvenlafaxine, tianeptine, vortioxetine, and mirtazapine. SSRIs may also be used in AD patients. TCAs were not regarded as safe agents for this indication. Regarding non-pharmacological approaches, the usefulness of exercise and psychological interventions based on social contact, reminiscence therapy, and cognitive rehabilitation was ratified, although their stand-alone use was not considered appropriate as an initial treatment.

The opinions summarized above mark a step forward and contribute to the field of depression in the context of dementia. They provide knowledge and clinical advice beyond limited CPG recommendations on diagnosis and treatment for such patients. They also give clarity regarding the most controversial etiological and clinical aspects of depression and dementia. However, future endeavors should continue addressing several unmet clinical needs, especially diagnostic criteria for depression and antidepressant tailored therapies in dementia patients in contrast to younger or cognitively healthy adults.

## Data Availability Statement

The raw data supporting the conclusions of this article will be made available by the authors, without undue reservation.

## Author Contributions

LA-O, RG-R, FG, JM, FO, JO, CP, and JP-E contributed equally to the study design and the development of the Delphi questionnaire. JL-Á prepared an extensive bibliography and analyzed and interpreted the results of the survey based on it. LA-O coordinated the group of experts. All authors contributed equally to the final manuscript.

## Conflict of Interest

LA-O has received grants from and served as consultant, advisor, or CME speaker for Janssen-Cilag, Exeltis, Lundbeck, Pfizer, Neuraxpharm, Sanofi-Aventis, and Servier. JO has received honoraria to participate in courses, lectures, congresses, and scientific advisory boards from several pharmaceutical companies including Exeltis, Janssen, Lundbeck, Angelini, Pfizer, Otsuka, and Esteve. JL-Á has prepared a monograph on this Delphi consensus with the financial help of Exeltis Pharmaceuticals Holding, S.L. JM has received grants from and served as consultant, advisor, or CME speaker for Almirall, Angelini, AstraZeneca, Bristol-Myers Squibb, Eli Lilly, Ferrer, GlaxoSmithKline, Janssen-Cilag, Lundbeck, Otsuka, Pfizer, Qualigen, Recordati, Sanofi-Aventis, Servier, and the Spanish Ministry of Science and Innovation (CIBERSAM). CP has received honoraria to participate in courses and lectures and has been invited to congresses and scientific advisory boards from several pharmaceutical companies including Exeltis, Janssen, Lundbeck, Angelini, Pfizer, and Casen. The remaining authors declare that the research was conducted in the absence of any commercial or financial relationships that could be construed as a potential conflict of interest.
